# Duality of the Brain—A Theory of Mind-Reading and Slate-Writing

**Published:** 1888-03

**Authors:** R. C. Word

**Affiliations:** Professor of Physiology in the Southern Medical College, Atlanta, Ga.


### Southern Medical Record.

A MONTHLY JOURNAL OF PRACTICAL MEDICINE.

☞All Communications must be addressed to R. C. WORD, M. D., Managing Editor.

"*Quicquid Præcipies Esto Brevis.*"

VOL. XVIII. ATLANTA, GA., MARCH, 1888. No. 3.

### ORIGINAL AND SELECTED ARTICLES.

### DUALITY OF THE BRAIN—A THEORY OF MIND-READING AND SLATE-WRITING.

BY R. C. WORD, M. D.,

Professor of Physiology in the Southern Medical College, Atlanta, Ga.

[*Read before the Medical Association of Georgia, April, 1887.*]

More attention has been given to the brain and nervous system of late years than at any former period in the history of physiological study. More has been dlscovered of nervous and mental phenomena in the last twenty or thirty years than was thought possible by our most lea:ned predecessors previous to that time.

The sensory and motor tracts to and from the cortex of the brain, and the points of decussation for motor and sensory impulses have been defined with at least an approximate certainty. Much has been accomplished in the study of automatic and reflex influences, in the localization of motor functions in separate and distinct regions of the brain, and in the location of numerous important nerve-centres.

Specialists in the treatment of nervous diseases are found in many places; journals in that department are being published, and the influence of the mind, as a powerful factor in the successful treatment of diseases, is attracting the attention, not only of the members of the profession, but also of the laity.

Among the late acquisitions to our knowledge of mental phenomena is the fact that we have two brains, each capable of independent action, and perfectly distinct, the one from the other.

A few years ago, in a public lecture in Washington City, Brown-Sequard discussed the question: "Have we two brains, and if we have, why not educate them?" He openly advocated the theory of two separate and independent brains in the human cranium, a theory which seems to conflict with the view commonly maintained, "that the left side of the brain is the exclusive organ serving to the movements and sensations of the right side of the body, and that the right side of the brain exclusively influences the left side of the body."

Dr. Wigan, of England, and also Sir Henry Holland, held a similar view in regard to the sufficiency of one hemisphere for the full performance of all the mental functions of the organ, and I well remember to have heard, long years ago, Professor Draper, of the New York Medical University, express a similar view on this subject.

Sequard, however, goes further than any other observer in the direction of the separate capacities of the two brains.

The fact is especially emphasized that an insane person sometimes knows he is insane; he knows he has insane ideas; he is rational and at the same time he is insane. Here it is claimed that one side of the brain acts normally and the other side abnormal y. In furtherance of this view of two brains, a case of a boy is mentioned at Notting Hill, London, who had two lives. "In the course of the day, generally at the same hour, but not constantly. his head was seen to suddenly fall. He remained erect, however, if he was standing, or if sitting he retained this position; if talking, he stopped for a while; if making a movement, he stopped moving. After continuing one or two minutes in this state of falling or dropping of the head, appearing as if asleep with his eyes closed, he would suddenly raise his head, open his eyes, being quite awake, and then ask, if there was anybody in the room whom he had not previously seen, who the person was, and why he was not introduced to him—being all the time in a state quite different from that of wakefulness. He had seen me many times (said Dr. S.) and knew me quite well. Being with him once when one of these attacks occurred, he lifted his head and asked his mother, "Who is that gentleman? Why don't you introduce him to me?" His mother introduced me. He did not know me at all. He shook hands with me, and then I had a conversation with him, such as a physician may have with a patient. In another instance, when with him again, while he had the same kind of attack, I

found that he recognized me fully, and talked of what he had spoken of in our first interview. I ascertained from what I witnessed myself, and from what I obtained from his mother, a very intelligent woman, that he had in reality two lives, two mental lives—one in his ordinary state and another occurring after those attacks of a kind of sleep for about a minute or two, when he knew nothing of what existed in his other state, in his ordinary life; that was all a blank. He knew nothing during that second state but what had occurred in previous periods of that same condition, but he knew full well all that had occurred then; and his recollection of everything was as perfect then as it was during his ordinary life concerning his customary acts of that state. He had therefore, as I have said already, two absolutely distinct lives, in each of which he knew everything that belonged to its wakeful period; and in neither of which did he know anything of what had occurred in the other. He remained in his abnormal state for a time, which was extremely variable, between one and three or four hours, and after that he fell asleep, and passed out of that state of mind pretty much in the same way that he had gone into it. I have seen three other cases of this kind."

Some years ago a case of this kind, which occurred at Watseka, in one of the Northwestern States, created a great sensation, and was called the "Watseka Wonder." The spiritualists claimed that the patient was alternately possessed, first by one spirit and then by another—the two states or conditions being very unlike, giving to the patient two lives, in which were manifested different actions and dispositions. In the one state there seemed to be no knowledge or recollection of facts or incidents which occurred in the other. These abnormal conditions would last sometimes for several days.

Other cases of the same kind are reported as having occurred in the observation of different practitioners.

Sequard does not controvert the generally admitted view that the right side of the brain presides over the left side of the body, and the left side of the brain over the right side of the body. He admits the fact and accepts it as a general rule, but meets it with a statement "that philosophers do not accept conclusions because there are some facts which support them," and gives facts which show different results. To instance: "Disease in one-half of the brain has been known to produce complete loss of sight of one eye, sometimes of the same side, sometimes of the opposite side." And then again: "Disease of one-half of the brain may exist without any effect upon the sight whatever." He holds "that an alteration in any part of the nervous system, whether in the brain or elsewhere, can, by producing an irritation, act on other parts so as to cause the loss of function in the part so acted upon, and that he has seen injuries of the spinal cord produce the loss of sight in the eye of the same side. Even the irritation of worms in children has been known to affect the power of sight. In the same way an irritation existing in the brain may produce the loss of function in another part, while it is known also that any part of one side of the brain may be diseased and the sight still remain good." The conclusion, then, would seem inevitable that one-half of the brain is sufficient for the preservation of the sense of sight on both sides, which tends to confirm the theory of the duality of the brain.

As regards voluntary muscular motions, though ordinarily controlled on the one side by the brain of the opposite side, it is affirmed that there are muscles in the neck, in the eye, in the throat and in the back also, which escape paralysis when one-half of the brain is diseased, a fact not to be explained on the old theory. Seven instances are mentioned of the destruction of one entire half of the brain without impairment of volitional movements on either side of the body. We cannot, therefore, look upon one-half of the brain as being necessarily or exclusively the organ serving to the movements of the body on the opposite side. It is possible, in some individuals at least, for one side of the brain to control voluntary movements in both sides of the body.

Touching recent observations in respect to cerebral localizations, of the faculty of speech on the left side, and other facts indicating special and peculiar powers in the one side or the other, it is urged that these differences depend upon education and development, and not upon any original difference in the two hemispheres.

Organs are developed in proportion to their use or exercise. Certain parts of the brain have been used for certain functions until they have become the better adapted to these functions.

A majority of people being right-handed shows the predominance of the left brain, which indeed receives more blood than the right side and is the largest. For some reason the right side of the body is most used, and the left side of the brain, which moves the right side, is more largely developed. Primitively it would seem that there was some natural cause for the greater development of the left brain. If persons are left-handed it indicates exceptional development in the right brain. These differences have been, perhaps in most instances, handed down by hereditary transmission. Facts are given which show the power of use in establishing and transmitting these peculiarities, and to show that they do not depend upon any original or primitive differences, but that either brain can be educated to perform, and has often exercised, all these functions.

The brain, then, is, to all intents and purposes, a dual organ, and if pains were taken in the training of children to exercise both brains, they could be equally developed in respect to every function and equally enlarged in any and every capacity.

This being true, though the two brains ordinarily act as a unit, they may, and sometimes do, act separately, and this possibly in cases wherin no disease exists in either hemisphere as a cause for non-action.

And here I take occasion to advance a theory original with myself: it is that under certain peculiar circumstances one side of the brain may converse with the other side.

This view, which at first thought will doubtless strike many as absurd or ridiculous, becomes more and more plausible as we investigate it, and serves as a hypothesis by which certain mysterious phenomena may be solved which hitherto have baffled every effort at explanation. I refer to mind-reading, slate-writing, etc.

Professors Carpenter, Oliver and other able physiologists attribute these mental operations to what they are pleased to call unconscious cerebration.

Sir William Hamilton writes of it as latent or concealed thinking. Leibnitz speaks of it as "the voluntary and involuntary mind;" another distinguished observer as "the conscious and unconscious mind."

Wendell Holmes says, "The more we examine the mechanism of thought the more shall we see that the automatic, unconscious mind enters largely into all of its processes."

Kant says, "We may become aware indirectly that we have an idea, although we be not directly cognizant of it."

Dr. Hazzard defines the mind to be that which thinks, feels and wills, and speaks of the self or Ego that takes cognizance of the thinking, feeling and willing. The Ego is but another name for consciousness, or what he elsewhere terms "Awakeness." "What we are awake to we are conscious of; that mind to which the Ego is not awake is the unconscious mind."*

While these and other able authorities have noted the fact of these two mental states, no one, so far as I am aware, has thought

*Mental Science Magazine.

of their possible dependence upon two separate and independent brains—the one ordinarily passive, the other active; the one ordinarily automatic, the other active or conscious. Under some circumstances both may act automatically, and possibly, at times of extraordinary shock or excitement, both may become positive and aroused into conscious and vigorous activity, thus accounting for those extraordinary cases in which in a brief moment of time all the scenes and latent memories of the past have rushed with inconceivable rapidity through the doubly-aroused and excited brain.

The phenomena of mesmerism were formerly denied and ridiculed, but are now generally admitted to be true. Its various phases have been presented under different names, as animal magnetism, electro-biology, electro-psycology, hypnotism, etc.

For convenience of description I will style the party who is mesmerized the mesmerizee. The party who puts him under the influence I will call the mesmerizer. The mesmerizee is in a passive or receptive condition, which I will term electro-negative. The mesmerizee, or the electro-negative individual, when fully brought under the influence, is under complete control of the mesmerizer. In this condition his will-power is in abeyance and he acts, thinks and feels as the mesmerizer wills him to act, think and feel. He is wholly passive, and his brain and entire nervous system receives its impressions from the mesmerizer.

There are various grades and phases of the electro-negative state, in some of which the party may not be wholly hypnotized.

There are many persons who are constitutionally electro-negative. Perhaps in any ordinary congregation twenty per cent. would be found electro negative, and could be easily mesmerized, a larger portion being females. Another portion of perhaps twenty per cent. would prove partially so, and by practice and perseverance could be also brought under the mesmeric influence; the remaining portion of the congregation, probably, either from the natural constitution of their nervous systems, or from skeptical opposition, could not at all be brought into the mesmerized state.

The more frequent one is thrown into the electro-negative state the more susceptible he becomes, and the more easily and readily he catches the thoughts and impressions of the mesmerizer, obeying either consciously or unconsciously the mandates of his will and reading his every thought and impression. There are those who, from constitutional proclivity and by much practice of entering into the electro negative state, acquire a very high degree of susceptibility to its influences. One may practice it until he is all the while in that relation to any one with whom he may come in contact. Such persons may even throw themselves into a hypnotic or trance condition.

The mind-reader, so-called, is an electro-negative subject highly developed, who has become so exceedingly susceptible that when in *rapport*, or nervous connection with another, he gets mental impressions or thoughts from him without difficulty, especially if the party whose thoughts he seeks to read concentrates his mind upon a particular object, by which he is made the more positive, and the nerve-force or magnetism emanating from him the more easily dominates or controls the other party who is passive, and recipient to both mental and physical impressions.

Now I go further, and hold that it is possible, in certain exceptional instances, for one side of the brain to be electro-positive and the other side electro-negative in the same individual. Under these circumstances the link or connection which ordinarily ties the two brains together is in some mysterious way severed, or for the time being deprived of its co-ordinating influence. This might occur from the reversal or shutting off of the usual or normal electric or nerve currents passing between the two hemispheres.

In this condition the electro-positive side may ask questions which may be automatically answered by the electro-negative side. Herein we find an explanation of what is called slate-writing practiced by what are called slate-writing mediums or spirit-writers.

Under these circumstances any incident or m mory which is latent in the brain is liable to be revived and to be automatically and unconsciously reproduced by the medium, and when thus presented comes with all the force and conviction of a communication from a third or an outside party.

Thus the slate-writer gets messages from his or her own brain, or if brought into *rapport* with another party may get mental impressions from him also.

*Rapport* does not, in every case, imply actual contact. The force or agency by which the phenomena is produced may or may not be electricity. It is claimed that the rate at which electricity travels is many thousand miles per second, while nerve force has been shown to travel not exceeding three hundred feet per second. Yet the argument is not deemed conclusive, as the velocity of electricity along a copper wire may far exceed that along the soft moist tissue of a nerve cord. At all events it may be safely assumed that nerve force, if not electricity, is in most respects analogous to it. We know that actual contact, though essential to the direct passage of an electric current from one conductor to another, does not prevent its diffusion in some degree to the surrounding media, especially upon conductors near by, as evinced by what is called induction, so often a disturbing element in our elephone communications.

The word *rapport* simply means a nervous relation or connection between the parties. If a circle be formed, consisting of a number of persons, the electro-negative subject may get mental impressions from any one of them.

For this, it would seem to be sufficient that only one side of the brain be in the passive or elect o-negative state, in which case the thoughts of the medium, as well as the thoughts of any member of the circle, are liable to be automatically written.

It is possible with some mediums that both sides of the brain may become electro-negative, in which case they pass into a hypnotic or trance condition, which is no other than the mesmeric state.

To those who deny the phenomena of slate-writing I have only to say that I personally know it is true, having witnessed it under such tests and under such varied circumstances as to place it beyond the shadow of a doubt. I, of course, admit that many have claimed to practice this power who were imposters and tricksters; but one single instance wherein the slate-writer has written automatically, again and again, carefully concealed written questions and unspoken mental questions correctly, even to the most hidden and secret thoughts of the questioner—I say one such writer is sufficient to establish the possibility and truth of the phenomena, and such an one I saw, and on many occasions satisfactorily tested.

Now, whether or not in the passive or electro-negative state of the human brain, in which mind so readily acts upon mind, an outside or departed spirit can also act upon and through the brain of the electro-negative subject, I do not here propose to discuss, nor do I deny its possibility.

The organist who uses a many-keyed instrument to evoke harmonious notes of music well illustrates, in our view, the human soul, which, in earth life, uses the machinery of the brain and nerves, with the five senses as media through which to impart thoughts and receive impressions from the material world; but on leaving its tenement of clay it cannot be supposed to see and communicate with the physical world without the machinery of the brain and nerves through which to operate. Having entered the spiritual realm, we suppose that its capacities, however much enlarged, are wholly spiritual. It probably possesses no power to communicate its thoughts to the inhabitants of earth, unless it be by entering a material body, and operating through the brain and physical senses of a living organism. Being immaterial in its nature and capable of permeating matter and solids of every kind, there seems to be no reason apparent why it might not enter and temporarily enthrone itself in the brain of an electro-negative subject, which is passive and offers no resistance to such occupancy.

In this case it may be supposed to hold the same relation to the party possessed as the mesmerizer holds to the mesmerizee, and could control his will, thoughts, feelings and imagination, causing him to utter speech, to write automatically, to hear clairaudiently, to see clairvoyantly, and to perceive or imagine materialized forms. It is strange, yet possibly it may be true.

> "There are more things in heaven and earth, Horatio,
> Than are dreamt of in your philosophy."

